# Quality and reliability of clear aligner information on Brazilian orthodontists’ websites: a DISCERN-based evaluation

**DOI:** 10.1590/2177-6709.31.3.e262657.oar

**Published:** 2026-09-28

**Authors:** Mohamad Jamal BARK, Gil Guilherme GASPARELLO, Rosilene Andrea MACHADO, Tayla Granemann JEDE, Matheus Melo PITHON, Isabela Alex KLOSS, Orlando Motohiro TANAKA

**Affiliations:** 1 Pontifícia Universidade Católica do Paraná, School of Medicine and Life Sciences (Curitiba/PR, Brazil).; 2 Research Unit of Population Health, University of Oulu (Oulu, Finland).; 3 Universidade Estadual do Sudoeste da Bahia, School of Dentistry (Jequié/BA, Brazil).

**Keywords:** Orthodontics, Clear aligners, Consumer health information, DISCERN, Internet, Patient decision-making, Brazil, Ortodontia, Alinhadores transparentes, Informação em saúde ao consumidor, DISCERN, Internet, Tomada de decisões, Brasil

## Abstract

**Objective:**

This study aimed to evaluate, using the DISCERN questionnaire, the quality, reliability, and decision-support capacity of aligner-related information published on orthodontists’ websites.

**Methods:**

A cross-sectional study was conducted in July 2024. Google (www.google.com.br) was searched using Portuguese-language keywords (*“tratamento ortodôntico”,* “*alinhadores”*, and “*ortodontia*”) in incognito mode from a Brazilian IP address. The first 130 non-sponsored websites were screened; 113 met the inclusion criteria. Two calibrated orthodontists independently evaluated each website using the validated Brazilian Portuguese version of the DISCERN instrument. Inter-rater reliability was assessed using the Intraclass Correlation Coefficient (ICC). Descriptive statistics and Spearman rank-order correlations were calculated using SPSS v.25.

**Results:**

The mean overall DISCERN score was 26.3 (SD = 5.8; range 15-52) out of a maximum of 75. The ICC was 0.87 (95% CI = 0.82-0.91), indicating good-to-excellent inter-rater reliability. Among the 113 websites, 54.3% were rated as very poor, 30.7% as poor, 11.5% as fair, 3.5% as good, and none as excellent. The lowest-scoring items were Q4 (sources of information, 99.1% very poor or poor), Q5 (publication date, 87.6%), Q8 (areas of uncertainty, 95.6%), Q11 (treatment risks, 98.2%), and Q12 (consequences of no treatment, 100%).

**Conclusions:**

The quality of clear aligner information on Brazilian orthodontists’ websites is critically poor. Orthodontists should provide evidence-based, transparent, and balanced content - including treatment risks, alternatives, and references - to support informed patient decision-making.

## RESUMO

**Objetivo:**

Este estudo teve como objetivo avaliar, por meio do questionário DISCERN, a qualidade, a confiabilidade e a capacidade de suporte à tomada de decisão das informações relacionadas aos alinhadores publicadas em *websites* de ortodontistas.

**Métodos:**

Foi realizado um estudo transversal em julho de 2024. O mecanismo de busca Google (www.google.com.br) foi utilizado com palavras-chave em português (“tratamento ortodôntico”, “alinhadores” e “Ortodontia”), em modo anônimo, a partir de um endereço IP brasileiro. Os primeiros 130 *websites* não patrocinados foram analisados, dos quais 113 atenderam aos critérios de inclusão. Dois ortodontistas calibrados avaliaram independentemente cada *website* utilizando a versão validada em português brasileiro do instrumento DISCERN. A confiabilidade interexaminadores foi avaliada por meio do Coeficiente de Correlação Intraclasse (ICC). Estatísticas descritivas e correlações de Spearman foram calculadas utilizando o software SPSS v.25.

**Resultados:**

A pontuação média global no DISCERN foi de 26,3 (DP = 5,8; intervalo 15-52), em um máximo possível de 75. O ICC foi de 0,87 (IC 95%: 0,82-0,91), indicando confiabilidade interexaminadores de boa a excelente. Entre os 113 *websites* avaliados, 54,3% foram classificados como muito ruins, 30,7% como ruins, 11,5% como regulares, 3,5% como bons, e nenhum como excelente. Os itens com menores pontuações foram Q4 (fontes de informação, 99,1% muito ruim ou ruim), Q5 (data de publicação, 87,6%), Q8 (áreas de incerteza, 95,6%), Q11 (riscos do tratamento, 98,2%) e Q12 (consequências da ausência de tratamento, 100%).

**Conclusões:**

A qualidade das informações sobre alinhadores transparentes em *websites* de ortodontistas brasileiros é criticamente baixa. Ortodontistas devem disponibilizar conteúdos baseados em evidências, transparentes e equilibrados, incluindo riscos do tratamento, alternativas terapêuticas e referências, para apoiar a tomada de decisão informada pelos pacientes.

## INTRODUCTION

The global expansion of internet access has fundamentally transformed how patients seek and process health information. In Brazil, 86% of the urban population is now connected to the internet, with a substantial proportion using it as a primary source of health-related information.^1^ Before the digital era, orthodontists typically acquired patients through professional referrals or social interactions. Today, however, websites and social media platforms have become the dominant first point of contact between patients and providers.^2^ This shift carries significant implications: while the internet holds the potential to empower patients in shared decision-making, it also creates a permissive environment for misinformation, unsubstantiated claims, and commercially motivated content that may mislead consumers.^3^ ^,^ ^4^

Clear aligner therapy (CAT) has experienced remarkable growth over the past two decades, driven in large part by aggressive direct-to-consumer (DTC) marketing campaigns by manufacturers.^5^ These campaigns frequently exaggerate treatment outcomes, omit contraindications, and downplay scientific limitations, creating unrealistic patient expectations.^6^ In parallel, orthodontists’ websites have become a critical channel for patient education and practice promotion. However, the dual role of these platforms, as both clinical information sources and marketing tools, creates an inherent tension between educational quality and commercial interest.^7^

The DISCERN instrument, developed by the University of Oxford, is the first validated, standardized tool for evaluating the quality of written consumer health information about treatment choices.^8^ It consists of 16 items organized into three sections: reliability of the source (items 1-8), quality of information about treatment choices (items 9-15), and an overall quality rating (item 16). Each item is scored on a 5-point Likert scale, with a maximum total score of 75 (excluding item 16). The DISCERN tool has been widely applied in orthodontic research across multiple countries and healthcare contexts.^9^ ^-^ ^12^ A validated Brazilian Portuguese version of the instrument is available, enabling its direct application in the Brazilian context.^13^

International studies have consistently documented poor quality of online orthodontic information. In the United Kingdom, Alsaqabi et al.^9^ evaluated websites advertising aligner therapy and reported a mean DISCERN score of 42.9 (SD = 9.4), with only 16% of websites rated as good or excellent and 31% as poor or very poor; only 11% fully complied with General Dental Council (GDC) ethical advertising guidelines. More recently, Nehra et al.^5^ conducted a comprehensive assessment of UK orthodontic appliance websites against Advertising Standards Authority (ASA) Committee of Advertising Practice (CAP) Code standards, finding that 95% of websites omitted common treatment risks, 92% failed to mention alternative treatments, and only 4% of claims were substantiated by evidence; inter-rater reliability was near-perfect (Fleiss’ kappa = 0.939). In the United States, Ferlias et al.^10^ evaluated 301 orthodontic websites and reported a mean DISCERN score of 36.78/80, reflecting poor to fair quality overall. Alpaydın et al.^11^ assessed 111 websites across multiple search engines and found a mean DISCERN score of 29.95/75, concluding that the quality of web-based information about clear aligners was poor. In Australia, Meade and Dreyer^12^ reported that the majority of DTC aligner provider websites scored poor or very poor on the DISCERN instrument, with particular deficiencies in items related to treatment risks and information bias.^13^ Patel and Cobourne^14^ evaluated 100 UK dental practice websites using both the DISCERN and LIDA instruments, reporting a mean DISCERN score of 48/75 (64%) and a mean LIDA score of 110/144 (76%), with the lowest performance in the reliability domain.^14^

Despite this growing international evidence base, no study has specifically evaluated the quality of clear aligner information on orthodontists’ websites in Brazil, a country with one of the largest orthodontic markets in the world and a highly digitally engaged population. Furthermore, the decision-making implications of poor online information are substantial: Crispino et al.^2^ found that 64.6% of prospective orthodontic patients searched the internet before their clinical visit, with Google being the primary platform (81%), and that patients who perceived online information as reliable were unlikely to discard it even when it contradicted professional advice. Similarly, studies of YouTube content on orthodontic topics have shown that even videos produced by orthodontists score poorly on quality assessments, lacking scientific rigor and omitting critical clinical information.^15^ ^,^ ^16^

The present study therefore aimed to evaluate, using the validated DISCERN instrument, whether the content published on Brazilian orthodontists’ websites provides high-quality, evidence-based, and reliable information regarding clear aligner therapy, and whether such content enables patients to actively participate in treatment decision-making.

## MATERIAL AND METHODS

### STUDY DESIGN

A cross-sectional, descriptive study was conducted to evaluate the quality and reliability of online information regarding orthodontic treatment with clear aligners published on Brazilian orthodontists’ websites.

### WEBSITE IDENTIFICATION AND SEARCH STRATEGY

An online search was performed using the Google search engine (www.google.com.br) on a desktop computer located in Brazil in July 2024. To minimize algorithmic personalization, the browser was operated in incognito (private browsing) mode, with search history, cookies, and cache cleared prior to each session. Geographic settings were configured to Brazil to ensure regionally relevant results. The following Portuguese-language keywords were used in separate searches: “*tratamento ortodôntico*” (orthodontic treatment), “*alinhadores*” (aligners), and “*ortodontia*” (orthodontics). These terms were selected to reflect the professional terminology most commonly used by orthodontists in their website content. The first 130 non-sponsored websites appearing in the search results for each keyword were identified for eligibility screening. This threshold was based on prior evidence that internet users rarely navigate beyond the first few pages of search results, meaning the top-ranked websites represent the content most accessible to prospective patients.^17^ Sponsored advertisements, Google Ads, and promoted listings were identified by their explicit labeling within the search results interface, and excluded from the sample. Duplicate domains were excluded, retaining only the first occurrence.

### INCLUSION AND EXCLUSION CRITERIA

Websites were included if they were written in Portuguese, discussed orthodontic treatment with clear aligners, and were maintained by individual orthodontists or private dental clinics located in Brazil. To confirm Brazilian origin, both the physical address of the practice and the professional registration of the dentist with a Brazilian Regional Dental Council (CRO) were verified. Websites were excluded if they were affiliated with corporate entities (e.g., aligner manufacturers or dental product companies), appeared as sponsored content, did not mention clear aligners among their orthodontic treatment options, or belonged to dental professionals who were not registered orthodontists. Websites belonging to orthodontic societies, academic institutions, or news portals were also excluded.

### SELECTION AND DATA EXTRACTION

Website selection and data extraction were independently performed by two calibrated orthodontists (Evaluators 1 and 2). Prior to data collection, both evaluators underwent a calibration session in which they independently scored a pilot set of 10 websites not included in the final sample. Discrepancies were discussed and resolved through consensus to standardize the application of the DISCERN instrument. In cases of disagreement during the main data collection phase, a third senior orthodontist was consulted to reach a final consensus. Each website was archived and evaluated at a single time point (T1 = July 2024) to ensure consistency in the dataset. Only textual content and its associated captions were analyzed; multimedia content (e.g., videos or infographics without accompanying text) was excluded from scoring.

### ETHICAL CONSIDERATIONS

This study involved the analysis of publicly available online content. No patient data, personal information, or human participants were involved. In accordance with national and international guidelines for research involving public domain data, formal ethics committee approval was not required for this study. The research was conducted in full compliance with the principles of the Declaration of Helsinki.

### ASSESSMENT INSTRUMENT

Each eligible website was evaluated using the DISCERN instrument, a validated tool developed by the University of Oxford to assess the quality of consumer-oriented health information about treatment choices.^8^ The Brazilian Portuguese version of the instrument was used, which has demonstrated excellent internal consistency and good reproducibility.^13^ The instrument consists of 16 questions, divided into three sections: Section 1 (items 1-8) evaluates the reliability of the publication and its sources; Section 2 (items 9-15) assesses the quality of information regarding treatment choices, risks, and alternatives; and Section 3 (item 16) provides an overall quality rating. Each item is scored on a 5-point Likert scale (1 = definitely no; 5 = definitely yes). The total DISCERN score was calculated by summing items 1-15, yielding a possible range of 15 to 75 points. Based on established scoring thresholds,^11^ websites were categorized as follows: Excellent (63-75), Good (51-62), Fair (39-50), Poor (27-38), and Very Poor (15-26).

### STATISTICAL ANALYSIS

Descriptive statistics (mean, standard deviation, minimum, maximum, and percentiles) were calculated to characterize the distribution of overall DISCERN scores and individual item scores. Inter-rater reliability between the two evaluators was assessed using the Intraclass Correlation Coefficient (ICC) with a two-way random-effects model and absolute agreement, reported with 95% confidence intervals (CIs). ICC values were interpreted according to the benchmarks proposed by Koo and Li^21^: values below 0.50 indicate poor reliability, 0.50-0.75 moderate reliability, 0.75-0.90 good reliability, and above 0.90 excellent reliability. Spearman rank-order correlation coefficients were used to evaluate associations between individual DISCERN items and total scores, given the ordinal nature of the data. A significance level of p < 0.05 was adopted for all analyses. All statistical analyses were performed using IBM SPSS Statistics, version 25.0 (IBM Corp., Armonk, NY, USA).

## RESULTS

### SAMPLE CHARACTERISTICS

A total of 130 websites were initially identified. After applying the inclusion and exclusion criteria, 113 websites met the eligibility requirements and were included in the final analysis. The excluded websites (n = 17) were removed for the following reasons: affiliated with aligner manufacturers or corporate entities (n = 7), not maintained by a registered orthodontist (n = 5), did not mention clear aligner therapy (n = 3), and duplicate domains (n = 2). The included websites were created by Brazilian orthodontists registered with a Regional Dental Council, operating private clinics across seven Brazilian states in the South, Southeast, and Northeast regions.

### INTER-RATER RELIABILITY

The ICC between the two evaluators was 0.87 (95% CI: 0.82-0.91), indicating good-to-excellent inter-rater reliability and confirming that the calibration process was effective and that the data collection was robust.

### OVERALL DISCERN SCORES

The mean overall DISCERN score (items 1-15) was 26.3 (SD = 5.8; range 15-52) out of a maximum of 75. Based on the established classification thresholds, 54.3% of websites were rated as very poor, 30.7% as poor, 11.5% as fair, 3.5% as good, and none achieved the minimum score required for an excellent classification. Collectively, 85% of the evaluated websites were classified as poor or very poor, indicating critical deficiencies in the quality of patient-directed information about clear aligner therapy (Table 1). The correlations among the 16 DISCERN items revealed positive and significant associations across nearly all criteria (r ranging from 0.41 to 0.78, p < 0.001). This pattern indicates high internal consistency, particularly among items related to information clarity (Q3_1- Q3_6) and source reliability (Q3_9-Q3_14). 

**Table 1:** Distribution of websites by DISCERN quality classification (n = 113).

Classification	Score range	n	%
Very Poor	15-26	61	54.3
Poor	27-38	35	30.7
Fair	39-50	13	11.5
Good	51-62	4	3.5
Excellent	63-75	0	0.0
Total		113	100.0

These findings reinforce that the evaluators applied the instrument consistently, suggesting good structural stability of the DISCERN tool in the context of social media publications. The Chord Diagram (Fig. 1) visually illustrates the positive correlations among the DISCERN items, highlighting a strong interrelationship between the criteria of clarity, reliability, and overall information quality.

**Figure 1:**
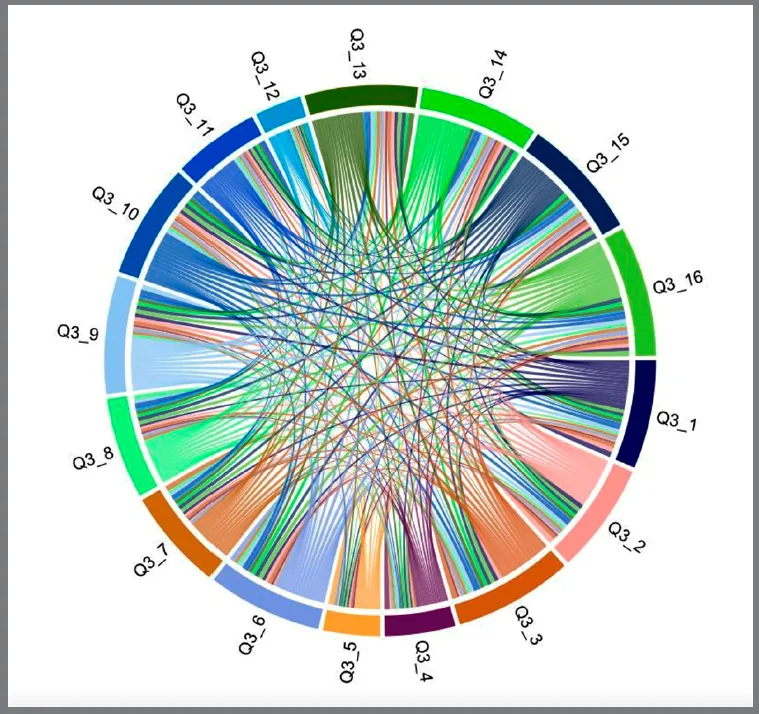
The Chord Diagram visually illustrates the positive correlations among the DISCERN items, highlighting a strong interrelationship between the criteria of clarity, reliability, and overall information quality.

### ITEM-LEVEL ANALYSIS

The mean scores for individual DISCERN items are presented in Table 2. In Section 1, which evaluates the reliability of the information source, the lowest-scoring items were: Q4 (*“Are the sources of information clearly stated?”*), with 93.8% of websites rated as very poor and 5.3% as poor (combined VP/P = 99.1%); Q5 (*“Is it clear when the information was produced?”*), with 80.5% rated very poor and 7.1% poor (combined VP/P = 87.6%); and Q8 (*“Does the text highlight areas of uncertainty?”*), with 87.6% rated very poor and 8.0% poor (combined VP/P = 95.6%).

**Table 2:** Mean DISCERN scores and percentage of very poor/poor ratings by item (n = 113).

Item	Description (abbreviated)	Mean (SD)	% VP	% P	% VP+P
Q1	Are the aims clear?	3.2 (1.1)	12.4	15.0	27.4
Q2	Does it achieve its aims?	3.0 (1.0)	14.2	18.6	32.8
Q3	Is it relevant?	3.4 (1.0)	8.0	12.4	20.4
Q4	Are sources of information stated?	1.1 (0.4)	93.8	5.3	99.1
Q5	Is it clear when information was produced?	1.2 (0.5)	80.5	7.1	87.6
Q6	Is it balanced and unbiased?	2.1 (0.9)	38.9	24.8	63.7
Q7	Does it provide additional sources?	1.4 (0.7)	71.7	15.0	86.7
Q8	Does it refer to areas of uncertainty?	1.2 (0.5)	87.6	8.0	95.6
Q9	Does it describe how treatment works?	2.8 (1.1)	20.4	22.1	42.5
Q10	Does it describe the benefits?	3.1 (1.1)	14.2	18.6	32.8
Q11	Does it describe the risks?	1.1 (0.4)	89.4	8.8	98.2
Q12	Consequences of no treatment?	1.0 (0.2)	98.2	1.8	100.0
Q13	Does it describe effects on quality of life?	1.8 (0.8)	53.1	27.4	80.5
Q14	Is there more than one treatment choice?	1.6 (0.8)	61.9	22.1	84.0
Q15	Does it support shared decision-making?	1.3 (0.6)	76.1	15.9	92.0
Q16	Overall quality rating	1.4 (0.6)	-	-	-

*SD = standard deviation; VP = very poor; P = poor. Q16 is not included in the total DISCERN score calculation.*

In Section 2, which assesses the quality of information about treatment choices, the most critical deficiencies were identified in: Q11 (*“Does the text describe the risks of each treatment?”*), with 89.4% rated very poor and 8.8% poor (combined VP/P = 98.2%); and Q12 (*“Does the text describe what would happen if no treatment were used?”*), with 98.2% rated very poor and 1.8% poor (combined VP/P = 100%).

### CORRELATION ANALYSIS

Spearman rank-order correlation analysis revealed statistically significant positive associations among the majority of DISCERN items (r ranging from 0.41 to 0.78, p < 0.001), indicating that websites scoring poorly on one dimension tended to score poorly across multiple domains. Notably, items related to source reliability (Q4, Q5) showed strong correlations with items assessing treatment risk disclosure (Q11, Q12) and shared decision-making support (Q15), suggesting that websites lacking transparency in their sources also tended to omit critical clinical information. The total DISCERN score was significantly correlated with all individual items, with the strongest associations observed for Q8 (r = 0.74), Q11 (r = 0.72), and Q15 (r = 0.71).

## DISCUSSION

The present study evaluated the quality of clear aligner information on 113 Brazilian orthodontists’ websites using the validated DISCERN instrument. The findings reveal a critical deficit: 85% of websites were rated as poor or very poor, with a mean DISCERN score of 26.3 (SD = 5.8) out of 75. None of the evaluated websites achieved an excellent classification. These results are consistent with, and in some respects more concerning than, findings from comparable studies conducted in other countries, underscoring the global nature of poor online orthodontic information quality.

In the United Kingdom, Alsaqabi et al.^9^ evaluated websites advertising aligner therapy and reported a mean DISCERN score of 42.9 (SD = 9.4), with only 16% of websites rated as good or excellent and 31% as poor or very poor. This marked discrepancy relative to our findings (26.3/75; 85% poor or very poor) may reflect genuine differences in professional standards, regulatory environments, and the influence of GDC ethical advertising guidelines in the UK context, which do not have a direct equivalent in Brazil. It may also partly reflect methodological differences, including sample selection and temporal differences in data collection. Patel and Cobourne^14^, also in the UK, reported a mean DISCERN score of 48/75 (64%) across 100 dental practice websites, with the lowest performance in the reliability domain - a finding that mirrors our results for items Q4, Q5, and Q8.

In the United States, Ferlias et al.^10^ reported a mean DISCERN total score of 36.78/80 across 301 orthodontic websites, reflecting poor to fair quality, with particular deficiencies in information about relapse risk and retention needs. Alpaydın et al.^11^ found a mean DISCERN score of 29.95/75 across 111 websites from multiple search engines, concluding that overall quality was poor. Meade et al.^18^ assessed 21 websites of marketed orthodontic products and found that 80.95% were categorized as poor quality, with a mean DISCERN score of 33.14 (SD = 5.44), and that only 8% of statements were objectively true. Meade and Dreyer^12^, evaluating DTC aligner provider websites in Australia, found that the majority scored poor or very poor on DISCERN, with particular deficiencies in items related to treatment risks and information bias. Collectively, these international data confirm that poor quality of online orthodontic information is a global phenomenon, not limited to any single country or regulatory context.

The most recent and comprehensive assessment of UK orthodontic appliance websites, conducted by Nehra et al.^5^ using the ASA CAP Code framework, found that 95% of websites omitted common treatment risks and 92% failed to mention alternative treatments. Notably, nearly 80% of websites used descriptive or vague language (e.g., “faster,” “painless,” “effective”) without quantitative substantiation, and only 4% of claims were supported by appropriate evidence. These findings are strikingly consistent with the present study’s results for Q11 (98.2% poor/very poor for risk description) and Q14 (84.0% poor/very poor for acknowledgment of treatment alternatives), suggesting that the deficiencies identified in Brazilian websites are not unique, but reflect a systemic pattern in how orthodontic treatment is marketed online, irrespective of national context.

The most critical deficiencies in the present study were concentrated in items related to source transparency (Q4, Q5), uncertainty acknowledgment (Q8), risk disclosure (Q11, Q12), and shared decision-making support (Q15). The near-universal failure to cite sources of information (Q4: 99.1% poor/very poor) and to indicate publication dates (Q5: 87.6% poor/very poor) renders the vast majority of these websites unreliable by the most basic standards of health information quality. These findings are particularly concerning given that 64.6% of prospective orthodontic patients search the internet before their clinical visit, with Google being the primary platform, and that patients who perceive online information as reliable tend not to discard it even when it contradicts professional advice.^2^

The almost universal failure to describe treatment risks (Q11: 98.2% poor/very poor) and the consequences of forgoing treatment (Q12: 100% poor/very poor) represents a significant patient safety concern. Manufacturers’ marketing campaigns frequently exaggerate treatment outcomes while downplaying contraindications, and orthodontists who reproduce or adopt similar promotional content without adding clinical nuance may inadvertently amplify misinformation.^5^ ^,^ ^6^ This is especially relevant in the context of Brazilian consumer protection law, under which joint liability may apply when a professional reproduces corporate marketing content that contributes to patient harm, even in the absence of direct misconduct.^19^

The low scores for Q8 (areas of uncertainty: 95.6% poor/very poor) and Q15 (shared decision-making support: 92.0% poor/very poor) indicate that the vast majority of websites do not acknowledge the limitations of clear aligner therapy or present information in a manner that supports autonomous patient decision-making. This is inconsistent with the ethical obligations of healthcare professionals and with the principles of informed consent, which require that patients be made aware of the benefits, risks, alternatives, and uncertainties associated with any proposed treatment.

The emergence of artificial intelligence (AI)-generated website content represents an additional and growing concern. AI-assisted content creation tools are increasingly used by dental practices to generate website text; however, these systems frequently draw upon existing online content, which may itself be of poor quality or commercially biased, potentially amplifying the dissemination of low-quality information.^20^ Orthodontic professional bodies and regulatory authorities should consider developing evidence-based guidelines for online content quality, analogous to the GDC ethical advertising standards in the UK or the ASA CAP Code, to provide practitioners with clear benchmarks and accountability mechanisms.

### LIMITATIONS

Several limitations of the present study should be acknowledged. First, the search was conducted using professional terminology (“*alinhadores*,” “*tratamento ortodôntico*,” “*ortodontia*”), which may have skewed the sample toward more professionally oriented websites and potentially excluded those most frequently accessed by lay patients using colloquial terms (e.g., *“aparelho invisível,” “aparelho transparente”*) or brand-specific names. Future studies should incorporate lay terminology to better capture the websites encountered by the general public. Second, the dynamic nature of online content means that websites may have been updated between the date of data collection (July 2024) and manuscript submission; websites were not rechecked prior to submission. Third, only textual content was analyzed; multimedia elements such as videos and infographics were excluded from scoring, which may have underestimated the informational value of some websites. Fourth, the study was limited to websites appearing in the top results of a single search engine (Google), and the influence of search engine optimization (SEO) and algorithmic ranking on the sample composition should be acknowledged as a potential source of bias. Fifth, the exclusion of corporate and manufacturer websites - although methodologically justified - means that the present study may have removed some of the primary sources of commercially driven misinformation; the low scores observed for individual orthodontists’ websites suggest that the problem extends beyond corporate actors. Finally, the absence of a predictive analysis limits the ability to identify factors associated with higher-quality websites, which would be a valuable direction for future research.

## CONCLUSION

The quality of clear aligner information on Brazilian orthodontists’ websites is critically poor, with 85% of evaluated websites rated as poor or very poor according to the DISCERN instrument, and a mean score of 26.3 (SD = 5.8) out of 75. The most significant deficiencies involve the absence of cited sources, lack of publication dates, failure to acknowledge clinical uncertainty, and near-universal omission of treatment risks and alternatives. 

These findings are consistent with international evidence from the UK, USA, and Australia, confirming that poor online orthodontic information quality is a global challenge. Orthodontists must assume responsibility for the quality of the health information they publish, ensuring that it is evidence-based, transparent, and designed to support - rather than undermine - informed patient decision-making.

## Data Availability

All data generated or analyzed during this study are included in this published article.
